# Effect of lycopene on titanium implant osseointegration in ovariectomized rats

**DOI:** 10.1186/s13018-018-0944-5

**Published:** 2018-09-17

**Authors:** Xiaojie Li, Wenli Xue, Yong Cao, Yanming Long, Mengsheng Xie

**Affiliations:** Department of Prosthodontics, College and Hospital of Stomatology, Guangxi Medical University, 10th Shuangyong Road, Nanning, 530021 China

**Keywords:** Osteoporosis, Osseointegration, Implant fixation, Lycopene, Ovariectomized, Biomechanical test

## Abstract

**Background:**

Lycopene prevents bone loss in osteopenic models. However, the role of lycopene in the success rate of dental implants under osteopenic conditions remains unknown. The aim of this study was to evaluate whether lycopene prevents delayed implant osseointegration in an ovariectomized (OVX) rat model.

**Methods:**

Thirty female Sprague-Dawley rats were randomly divided into the following groups: OVX with vehicle (OVX group), OVX with lycopene (OVX + lycopene group) and sham-operated with vehicle (sham group). Twelve weeks after ovariectomy or sham operation, titanium implants were placed into the distal metaphysis of the bilateral femurs of each rat. These rats were subsequently gavaged with lycopene (50 mg/kg/day) or vehicle. After 12 weeks of gavage, all rats were sacrificed, and specimens were harvested. Sample osseointegration was evaluated by biomechanical testing, 3D micro-computed tomography (micro-CT) analysis and histomorphometric analysis.

**Results:**

Compared with the OVX group, the OVX + lycopene group showed a 69.3% increase in the maximum push-out force (*p* < 0.01). Micro-CT data for the femurs in the OVX + lycopene group showed significantly higher bone volume, trabecular thickness and less trabecular space than did those in the OVX group. The bone area (BA) around the implant and bone contact (BC) with the implant were increased by 72.3% (*p* < 0.01) and 51.4% (*p* < 0.01) in the OVX + lycopene group, respectively, compared with those in the OVX group. There was no significant difference in the mechanical test, micro-CT scanning and histomorphometric data between the OVX + lycopene and sham groups (*p* > 0.05).

**Conclusions:**

Lycopene improved implant osseointegration, fixation and bone formation under osteopenic conditions, suggesting that lycopene is a promising therapeutic agent to prevent delayed implant osseointegration and bone loss under osteopenic conditions.

## Background

Implant-based treatment has been widely used in the fields of orthopaedics and dentistry. Regarding patient characteristics, aside from smoking and periodontitis as negative risk factors [1], the successful fixation and stability of dental implants depend on the implant site (mandible vs. maxillary) [2, 3] and bone quality and quantity around the implant [4]. Osteoporosis is a systemic skeletal metabolic disease characterized by low bone mass and micro-architectural bone deterioration, with a high risk of fragility fracture [5]. Although osteoporosis is not considered as a risk factor for dental implant failure [6, 7], initial implant stability can be influenced by both local and skeletal bone densities, and osteoporotic patients need more healing time [8, 9]. Proper implant osseointegration, i.e. direct contact between the implant and surrounding bone, is desired for mechanical fixation of the implant [10]. In a rat model, the quantity and quality of osseointegration remained significantly impaired under osteopenic conditions12 weeks after implantation, leading to decreased implant fixation [11]. Various therapeutic agents have been investigated and verified to be effective in promoting implant osseointegration in osteopenic bone [12]. Although adverse events are rare among approved osteoporosis treatment agents, in some cases, bisphosphonates and denosumab could cause osteonecrosis of the jaw, raloxifene may increase the risks of thrombus and stroke and teriparatide may cause nausea and other side effects [13]. Thus, new safe, cost-effective drugs that can accelerate implant osseointegration under osteoporotic conditions are still in demand.

The bone remodelling process is similar between osseointegration and fracture healing [10]. Some researchers have proposed that oxidative stress and antioxidants might play a role in fracture healing [14, 15]. Immediately after fracture, oxidative stress significantly increases due to severe bone loss and ischaemia [14]. Reactive oxygen species (ROS) can cause cell DNA damage, accumulate in the injury site and have detrimental effects on bone healing [15]. During normal healing, the antioxidants in the body increase correspondingly to scavenge excessive ROS [14]. However, in cases when the antioxidant system is compromised, elevated ROS activity leads to oxidative stress, which inhibits osteogenesis and can reduce bone regenerative capacity [15]. Thus, an imbalance between excessive ROS generation and an insufficient antioxidant defence mechanism makes these fractures difficult to heal. Bednarek-Tupikowksa et al. demonstrated higher oxidative biomarkers and reduce d antioxidative potency in postmenopausal women than in premenopausal women [16]; these findings are associated with compromised bone healing capacity due to osteoporosis. However, antioxidant treatment promoted bone healing in rats with severe bone loss [17]. Furthermore, an antioxidant-based diet prevented bone loss in menopausal women [18], and a case-control study showed that the antioxidant intake score was inversely associated with hip fracture risk in ever-smokers but not in never-smokers [19]. Therefore, antioxidant therapy might be useful in promoting osseointegration under osteoporotic conditions.

Lycopene, a carotenoid found in red fruits and vegetables, is one of the most potent natural antioxidants and has the highest scavenging capacity for singlet oxygen molecules among all carotenoids [20]. Lycopene has been used for decades to prevent chronic diseases [21]. Lycopene exerts its antioxidant effect mainly through activating the antioxidant response element/electrophile response element (ARE) transcription system by disrupting the cytosolic interactions between the major ARE-activating transcription factor, nuclear factor erythroid 2-related factor 2 (Nrf2) and its inhibitor, Kelch-like ECH-associated protein 1 (Keap1) [22]. Recently, Mackinnon et al. showed that a lycopene-restricted diet significantly decreased circulating lycopene and decreased biomarkers of oxidative stress and bone resorption in healthy postmenopausal women [23]. In addition, in a pilot study using a small group of healthy postmenopausal women, the same group showed that lycopene supplementation significantly decreased the detection of oxidative stress markers and a bone resorption marker [24]. These findings suggest that lycopene has a positive effect on bone metabolism by reducing the rate of bone resorption. Interestingly, a prospective cohort study of 946 men and women reported that lycopene intake is inversely associated with the risk of osteoporotic hip fracture [25]. Moreover, lycopene supplementation prevents bone loss and maintains bone strength in ovariectomized (OVX) rats [26]. However, the role of lycopene in implant osseointegration under osteopenic conditions remains unknown.

OVX rats have been widely used as a model of postmenopausal osteoporosis in humans [27]. OVX rats have also been used in many studies on implant osseointegration under osteopenic conditions [12]. Implant osseointegration could be achieved in OVX rats, but according to histomorphometric and functional analysis, less osseointegration occurred in OVX rats than in sham rats within 2–12 weeks [11, 28, 29]. These results indicated that a longer healing time was needed due to compromised healing capacity under osteopenic conditions, which is similar to the implant healing process of humans.

We hypothesized that lycopene prevents delayed implant osseointegration under osteopenic conditions. In our study, we used OVX rats as an osteopenic model and analysed the effect of lycopene on titanium-based implant osseointegration.

## Methods

### Implant preparation

Sixty titanium cylindrical implants (2.5 mm in diameter and 4 mm in length) were custom-made of TA3 pure titanium (Fox Ti Tech, Shanghai, China). The surfaces of the implants were blasted with aluminium oxide to obtain a rough appearance without any coating. Next, the implants were washed three times with distilled water, sonicated for 10 min in high concentrations of trichloroethylene and ethanol, autoclaved at 134 °C and under 0.205 MPa for 8 min and stored in a drying cabinet.

### Animals

Thirty female Sprague-Dawley rats (Animal Centre of Guangxi Medical University), aged 12 weeks old with a weight of 245 ± 7.46 g, were used in this study. Animals were given a standard laboratory diet and tap water and were maintained under climate-controlled conditions (25 °C; 55% relative humidity; 12 h light and 12 h darkness). All procedures followed were in accordance with the ethical standards of Animal Care and Use of Guangxi Medical University. The study protocol was approved by the Animal Experimental Ethical Committee of Guangxi Medical University (ID Number: 201601007).

### Ovariectomy and implantation procedures

After 1 week of acclimatisation, 30 rats were randomly divided into the following groups: OVX with vehicle (OVX group, *n* = 10), OVX with lycopene (OVX + lycopene group, *n* = 10) and sham-operated with vehicle (sham group, *n* = 10). Rats in the OVX group and OVX + lycopene group underwent bilateral ovariectomy following a previously described procedure [30], and ten rats underwent sham surgery (sham group). After surgery, the rats were fed with standard rat chow for 12 weeks.

**Fig. 1:**
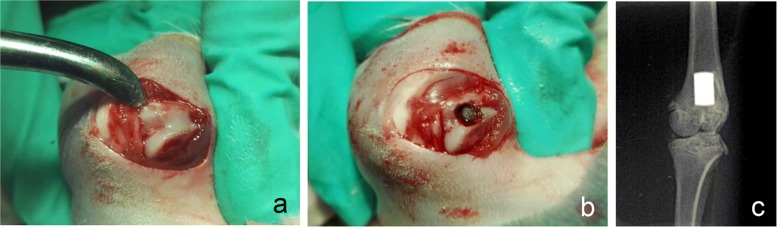
The operative location of the implant. **a** The exposed knee joint. **b** An implant was inserted into the channel. **c** Radiograph of the implant in the distal metaphysis of femur after surgery

Twelve weeks after ovariectomy, titanium implants were inserted bilaterally into the distal femur using a previously described procedure [30]. Briefly, the rats were anaesthetized by intraperitoneal injection of 10% chloral hydrate (3.0 ml/kg; Kelon Biosciences, Chengdu, China) and were incised above the knee of the hind limb to expose the knee (Fig. 1a). A channel was drilled from the intercondylar notch to the medullary canal in a longitudinal direction parallel to the long axis of the femur using a straight fissure bur (2.0 mm in diameter) with a speed of 800 rotations per minute (RPM). Cold (4 °C) saline irrigation was applied during the entire drilling procedure. Titanium implants were inserted into the channels with a titanium mallet to a position at which the distal implant surface was approximately 1 mm below the cartilage surface (Fig. 1b, c). The incision was then closed with sutures. For pain control, subcutaneous injection of 5% carprofen (0.3 ml/kg; Vland Biotech, Qingdao, China) was administered for 2 days following the procedure.

### Drug intervention

Ardawi et al. [26] reported that lycopene prevented bone loss in OVX rats in a dose-dependent manner. In their study, the highest dosage of the OVX + lycopene group, which was 45 mg/kg/day, showed the best effect on bone mass in OVX rats. Therefore, for easy calculations, a dosage of 50 mg/kg/day, which is closest to the highest dosage used in their study, was chose0n. After implantation surgery, rats in the OVX + lycopene group were gavaged with lycopene (50 mg/kg/day, Hezhong Biosciences, Wuhan, China) dissolved in corn oil (10 mg lycopene/ml) for 12 weeks, while rats in the OVX and sham groups were gavaged with corn oil (5 ml/kg/day). During the 12-week gavage period, three rats died (one in each group), possibly due to oesophageal damage. Twelve weeks after implantation, the rats were sacrificed, and both femurs were harvested. For each rat, the left femur was used for histological analysis, and the right femur was used for biomechanical testing and micro-computed tomography (micro-CT) analysis. For each group, nine specimens were used for histological analysis, and nine specimens were used for mechanical testing and micro-CT analysis.

### Biomechanical testing

**Fig. 2:**
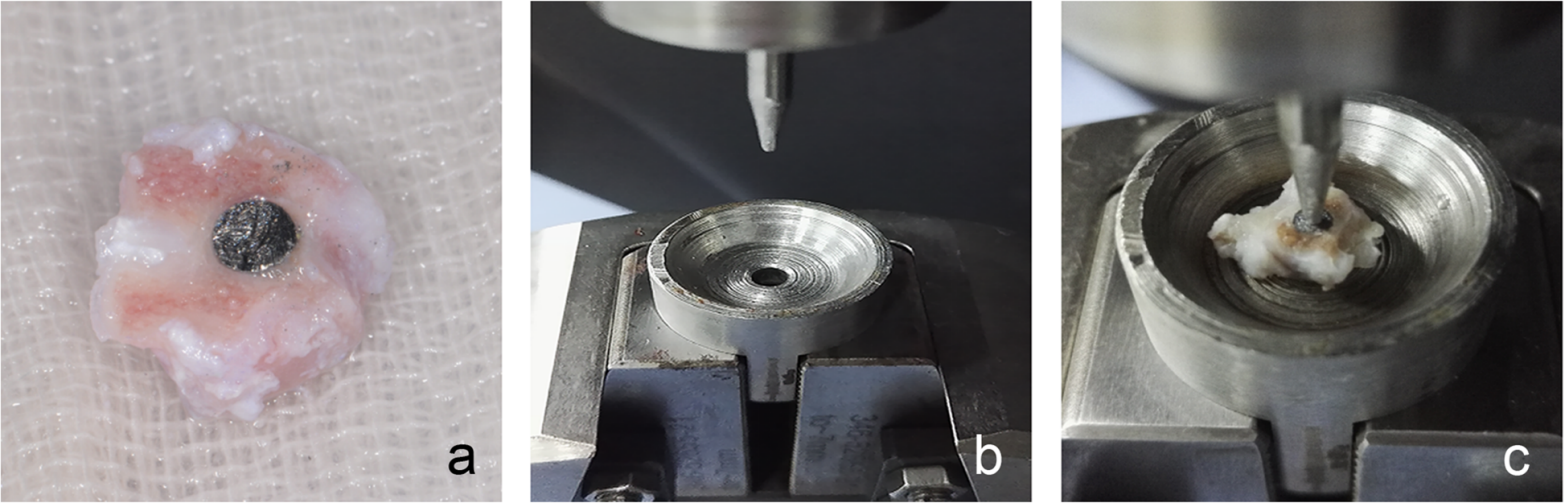
Photographs of the mechanical test set up. **a** A specimen with an implant. **b** The mechanical test machine bench with the jig. **c** A specimen being mechanically tested

Immediately after sacrifice, the femurs were trimmed to expose both ends of the implant (Fig. 2a) with a high-speed dental handpiece and a diamond bur (SF-12C, Mani, Japan) without damaging the implants. By placing the distal end of the specimen on a flat surface to check whether the sample was evenly placed, the excess bone tissue was trimmed, and a flat specimen surface level to the distal end of the implant was obtained to increase the stability during the mechanical test. Samples were wrapped in gauze soaked with saline and were then stored at − 20 °C. Three days later, after all of the samples were prepared, they were thawed at 4 °C overnight and then delivered on ice for biomechanical testing. The biomechanical properties of bone-implant interfaces were tested by the push-out test method [30] using a universal mechanical testing machine (AGS-X; Shimadzu, Kyoto, Japan). The specimen was evenly placed on a jig hole 0.5 mm larger than the implant diameter (Fig. 2b), with the proximal end facing upwards. The push-out rod was moved as close to the specimen as possible without touching it. Next, a vertical force (parallel to the longitudinal axis of the implant) with a displacement speed of 0.5 mm/min was applied to the implant (Fig. 2c). The test was stopped when the implant was completely free from the bone. The peak force was then recorded in Newtons (N).

### Micro-CT evaluation

In our pilot test, we scanned the sample with the implant before the push-out test and found that there were many metal artefacts that appeared as streaks or shadows, which compromised the readability of the image around the implant. To avoid metal artefacts, we gently removed the implants after mechanical testing and stored the bone tissue in 70% ethanol for micro-CT detection. The specimens were scanned using a micro-CT scanner (60 kV, 667 μA; ZKKS-MCT-Sharp; Kaisheng Technology, Guangzhou, China) at a high resolution and an isotropic voxel size of 20 μm. The scanning axis was along the axis of the femur. ZKKS-Micro-CT.3.0 software was used for 3D reconstruction and for 3D and quantitative evaluation. For the analysis of peri-implant bone tissue, the volume of interest (VOI) included the trabecular compartment extending 250 μm from the surface of the implant, assuming that the implant had not been removed. An equally long VOI was chosen for all samples, including 100 slices (2000 μm) beneath the lowest part of the growth plate, but the cortical bone was excluded semi-automatically if it was within the VOI. The bone volume/total bone (BV/TV) ratio, bone mineral density (BMD), trabecular thickness (Tb.Th), trabecular separation (Tb.Sp) and trabecular number (Tb.N) were calculated.

### Histological evaluation

Samples were trimmed with a high-speed handpiece and a diamond bur (SF-12C, Mani) to expose both ends of the implants. Then, the femurs with implants were fixed in 10% neutral formalin for 48 h and decalcified with 10% neutral ethylene-diamine-tetraacetic acid for 30 days. Next, the whole samples were placed on the mechanical test jig with the proximal end facing upwards, and the implants were gently pushed out along the axis of the implant with the push-out rod by hand. Specimens without implants were then dehydrated in graded ethanol and embedded in paraffin. Transverse 5-μm-thick sections were cut with a paraffin ultra-thin slicer (RM2235; Lecia, Heidelberger, Germany). The section at the growth plate level was selected as the section of interest and was stained with haematoxylin-eosin (HE). Histomorphometry was performed to semi-quantify the percentages of the bone area (BA) and bone contact (BC) with the implant for each sample using a semi-automated digital image analysis system, consisting of an optical microscope (BX61VS; OLYMPUS, Tokyo, Japan), OLYMPUS VS-ASW image collecting software and Image-Pro Plus 6.0 software (Media Cybernetics, Rockville, USA). Assuming that the implant had not been removed, BA was defined as the percentage area of the bone tissue found within an annulus of 0.25 mm around the implant to the total area of the annulus [31], while BC was defined as the percentage length of the direct bone-implant interface to the total implant surface [32].

### Statistical analysis

Data are expressed as the mean ± standard deviation (SD). Statistical analyses were performed with the SPSS 17.0 statistics package, using one-way analysis of variance (ANOVA) to compare three groups and the Student-Newman-Keuls test to perform multiple comparisons between two groups. A *p* value below 0.05 was set as the significance level.

## Results

### Biomechanical test

**Fig. 3:**
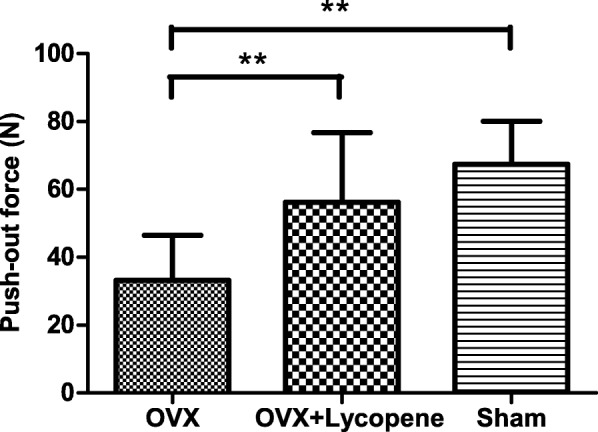
Quantitative result of the biomechanical test of implant fixation presented as the maximal push-out force. Error bars in the figures represent the SD, **p < 0.01 vs. the OVX group

Biomechanical testing data indicated that implant fixation decreased in the OVX group, while lycopene treatment almost prevented this effect. Compared with the sham group, the OVX group showed a 50.8% (*p* < 0.01) decrease in the maximum push-out force, while compared with the OVX group, the OVX + lycopene group showed a 69.3% (*p* < 0.01) decrease. There was no significant difference in the push-out force between the OVX + lycopene and sham groups (*p* > 0.05) (Fig. 3).

### Micro-CT analysis

**Fig. 4:**
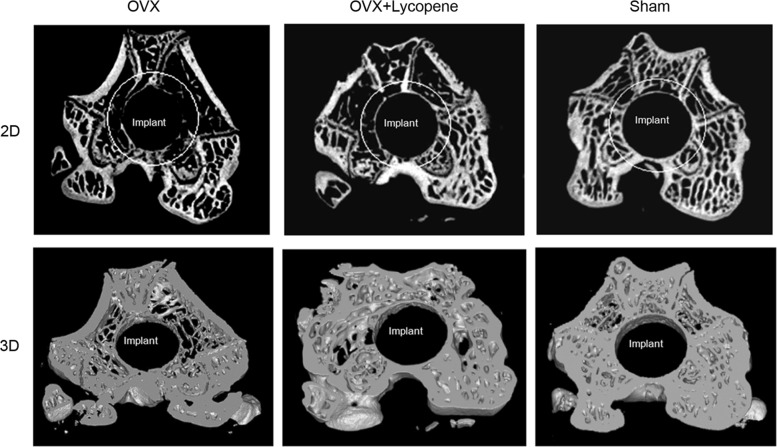
2D and 3D transverse micro-CT images of the distal metaphysis of femurs from the OVX, OVX + lycopene and sham groups

**Fig. 5:**
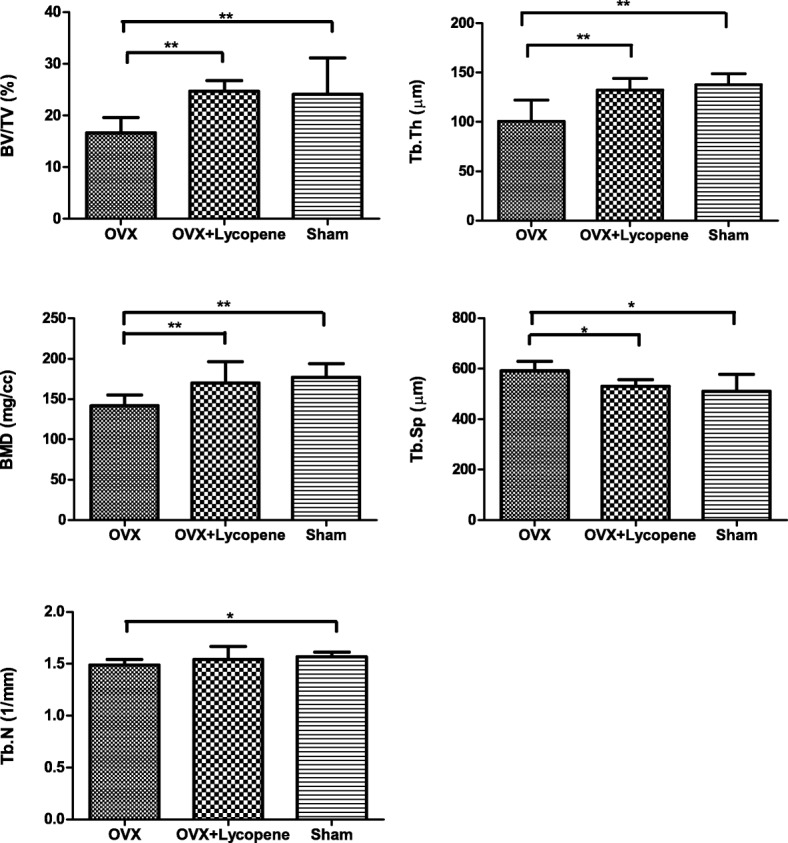
Quantitative results of peri-implant bone volume and trabecular micro-architecture by micro-CT analysis. BV/TV, percent bone volume; Tb.Th, mean trabecular thickness; BMD, bone mineral density; Tb.Sp, mean trabecular separation; Tb.N, mean trabecular number. Error bars in the figures represent the SD, *p < 0.05 and **p < 0.01 vs. the OVX group

In 2D and 3D transverse micro-CT images, the peri-implant bone volume was lower in the OVX group than in the sham group, while the volume was higher in the OVX + lycopene group than in the OVX group (Fig. 4). In the quantitative analysis of the peri-implant bone volume and trabecular micro-architecture within VOI, compared with the sham group, the OVX group showed a 33.1% decrease in the BV/TV ratio (*p* < 0.01), 26.9% in Tb.Th (*p* < 0.01), 20.0% in BMD (*p* < 0.01) and 5.1% in Tb.N (*p* < 0.05) (Fig. 5). Interestingly, compared with the OVX group, the OVX + lycopene group showed a 45.8% increase in the BV/TV ratio (*p* < 0.01), 31.5% in Tb.Th (*p* < 0.01), 20.0% in BMD (*p* < 0.01) and 3.4% in Tb.N (*p* > 0.05). Tb.Sp was 16.7% (*p* < 0.01) higher in the OVX group than in the sham group and was 7.7% (*p* < 0.05) lower in the OVX + lycopene group than in the OVX group. There was no significant difference between the OVX + lycopene and sham groups in BV/TV, BMD, Tb.N, Tb.Th and Tb.Sp (*p* > 0.05) (Fig. 5).

### Histological analysis

**Fig. 6:**
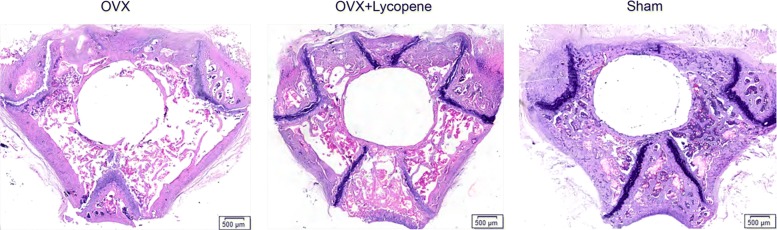
Light microscope images of transverse histological sections at the epiphyseal plate level of the distal metaphysis of femurs from the OVX, OVX + lycopene and sham groups. HE staining

**Fig. 7:**
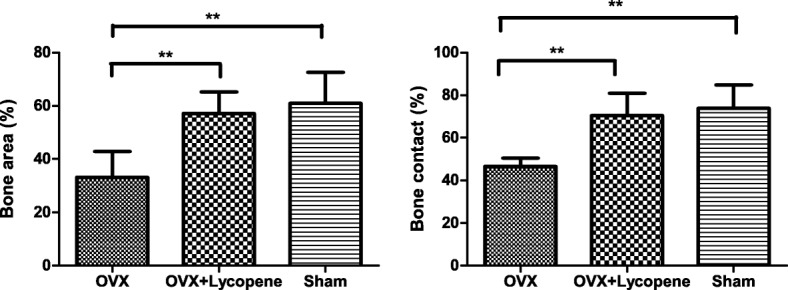
Quantitative results of histomorphometric analysis presented as bone area (BA) around the implant and bone contact (BC) with implant, assuming that the implant had not been removed. Error bars in the figures represent the SD, *p < 0.05 and **p < 0.01 vs. the OVX group

The decalcified sections showed the destructive effects of OVX surgery on implant osseointegration and peri-implant bone mass, while those values were increased in the OVX + lycopene group (Fig. 6). In quantitative analysis within a region of interest (ROI), compared with BA and BC in the sham group, BA and BC in the OVX group decreased by 45.6% (*p* < 0.01) and 37.0% (*p* < 0.01), respectively. However, BA and BC were 72.3% (*p* < 0.01) and 51.4% (*p* < 0.01) higher in the OVX + lycopene group than in the OVX group, respectively. There was no significant difference in BA and BC between the OVX + lycopene and sham groups (*p* > 0.05) (Fig. 7).

## Discussion

Osteoporosis, a common disease in elderly people, is characterized by low BMD and loss of structural and biomechanical properties [33]. The compromised bone healing capacity under osteopenic conditions shows adverse effects on implant fixation and osseointegration 2–12 weeks after implantation [11, 28, 29] and affects the initial stability and healing time of the implant in osteoporotic patients [8, 9]. Implant fixation can be mostly explained by the degree of osseointegration [34]. In this study, we histomorphometrically and functionally investigated the effects of lycopene on implant osseointegration in osteopenic rats. As expected, the sham group had more mechanical implant fixation, BC and bone mass than did the OVX group, indicating the destructive effect of osteopenia on implant osseointegration. Furthermore, as expected, lycopene maintained mechanical implant fixation, BC and bone mass around the implant in the OVX + lycopene group to almost the same level as that in the sham group. These results indicate that lycopene could prevent bone loss and maintain osseointegration in OVX rats 12 weeks after implantation. These findings suggest that lycopene might be a new therapeutic agent for preventing delayed osseointegration under osteopenic conditions.

The OVX rat model is most commonly used to simulate postmenopausal osteoporosis [35]. In our study, compared with sham rats, OVX rats showed significantly decreased BMD and compromised bone tissue micro-structure surrounding the implant. These results indicate that OVX can induce osteopenia in rats, in accordance with published data on the effects of OVX on bone mass [11]. In addition, osseointegration, implant fixation and micro-structure of the bone surrounding the implant were significantly lower in OVX rats than in sham rats. These results were in accordance with those of other studies [11, 32, 36, 37], indicating the adverse impact of OVX on osseointegration in rats.

Lycopene is a natural potent antioxidant that is considered a human nutritional supplement by the United States Food and Drug Administration (US FDA) [38]. The recommended dosages for humans are 5–7 mg/day to prevent chronic diseases and 35–75 mg/day to treat cancer and cardiovascular diseases [21]. The largest dosage reported for rats was 3000 mg/kg/day for 13 weeks without any adverse effects observed [39]. The only side effects in cases of excessive intake of lycopene were alterations in the skin and liver colouration, which were non-toxic and reversible [40]. Ardawi et al. [26] demonstrated the dose-dependent effect of lycopene on bone loss prevention in OVX rats. In their study, the highest dosage of the OVX + lycopene group, which was 45 mg/kg/day, showed the best effect on bone mass in OVX rats. In our study, 50 mg/kg/day lycopene was given to OVX rats by gavage for 12 weeks, and no adverse effects were observed. Therefore, lycopene is a relatively safe medication.

A number of studies have demonstrated the beneficial effects of lycopene on bone metabolism [23, 24, 26, 41–43]. In vitro studies have shown that lycopene promotes proliferation and differentiation of osteoblasts [26, 44] and suppresses osteoclastogenesis and differentiation of osteoclasts in normal and OVX rats [26, 45]. Lycopene treatment can increase the expression of bone formation biomarkers in rats [42] and can decrease the expression of bone resorption biomarkers in osteoporotic patients [24, 41]. Taken together, these findings indicate that lycopene has both anabolic and anti-catabolic effects on bone metabolism. In our study, histomorphometric and micro-CT analyses showed significantly increased BA, BMD and the micro-structure of the bone surrounding the implants in OVX rats, demonstrating the treatment effects of lycopene on local osteopenic bone. Our findings were consistent with the results of Ardawi et al. [26] and Iimura et al. [43], which both reported that lycopene prevented bone loss in OVX rats. Therefore, lycopene could be an effective therapeutic agent for osteoporosis.

Several drugs reportedly promote osseointegration and implant fixation by improving the micro-structure of the bone surrounding the implants in rats [36, 37, 46]. At the tissue level, osseointegration could be affected by the quantity and quality of the bone around the implant. Li et al. [11] reported impaired implant osseointegration and fixation in OVX rats with compromised peri-implant bone micro-structure. In our study, lycopene increased BA, BV/TV, BMD and Tb.Th and decreased Tb.Sp of peri-implant bone in OVX rats. Consistent with the results of the aforementioned studies, these results revealed that the role of lycopene in osseointegration might be mediated by altering the peri-implant bone mass and trabecular micro-architecture.

Our study has several limitations. First, the experimental situation is not representative of clinical situations. Second, measuring the decalcification of bone tissue might not be the best method for performing a histological analysis of the osseointegration rate. The interfaces of the bone remained intact by visual inspection after the implants were pushed out from the femur, as they did after embedding and sectioning. One possible reason why the interface between the implant and adjacent tissues was not apparently damaged is that the surface of the implant had no threads and was basically smooth. Another reason is that damage might occur only at small interfacial points and did not affect the calculation of BA and BC. However, the bone tissue on the slides was deformed to different degrees during the process of staining, possibly because the decalcified bone tissue was prone to exfoliation from the slides, making the inner boundary of the bone irregular and complicating the calculation of BA and BC. Based on this study, applying non-decalcified bone to the slide to maintain the original shape of the bone for better analysis of the osseointegration rate might be more appropriate. Third, we did not examine the condition of the bone at the time of implant placement. Lycopene-treated rats had better osseointegration than did OVX + vehicle rats at 12 weeks post-implant placement. This difference may have occurred due to the prevention of further bone loss between 12 and 24 weeks post-OVX or through a positive effect of lycopene on the healing process. Because we did not examine the condition of the bone at the time of implant placement, we cannot know whether lycopene improved or maintained osseointegration in the lycopene-treated group. Fourth, we did not record the uterine weights or examine oestrogen production levels before and after lycopene treatment (lower body weight was observed in lycopene-treated rats than in OVX + vehicle rats after 12 weeks of gavage, but the difference was non-significant; data not shown). Several authors have found that oestrogen production is stimulated by treatment with lycopene and other carotenoid derivatives [42, 47]. This stimulation is reflected in uterine hypertrophy [26] and could be responsible for the positive bone effects of lycopene treatment, making the effects of lycopene indirect. Due to a lack of recording of the variation in uterine weights and oestrogen production levels before and after the treatment, we cannot determine whether lycopene performed its role through oestrogen production. If lycopene’s mode of action is through oestrogen, then it will not have similar effects on male rats or non-OVX-induced osteopenic rats. Therefore, the specific mechanism of how lycopene promotes osseointegration under osteopenic conditions needs further study.

## Conclusions

We evaluated the influence of systematically administered lycopene on implant osseointegration in an OVX rat model. The results indicated that lycopene significantly increased implant osseointegration, fixation and bone mass in OVX rats to the level of those in sham rats. Within the limitations of the study, lycopene may be an effective medication for preventing delayed osseointegration under osteopenic conditions. Further research is warranted to determine the specific mechanism of how lycopene prevents delayed osseointegration under osteopenic conditions.

## Abbreviations

ARE: Antioxidant response element/electrophile response element
BA: Bone area
BC: Bone contact
BMD: Bone mineral density
BV/TV: Bone volume/total bone
HE: Haematoxylin-eosin
Keap1: Kelch-like ECH-associated protein 1
micro-CT: Micro-computed tomography
OVX: Ovariectomized
ROS: Reactive oxygen species
Tb.N: Trabecular number
Tb.Sp: Trabecular separation
Tb.Th: Trabecular thickness
VOI: Volume of interest
